# High spatial resolution inorganic scintillator detector for high‐energy X‐ray beam at small field irradiation

**DOI:** 10.1002/mp.14002

**Published:** 2020-01-23

**Authors:** Sree Bash Chandra Debnath, Carole Fauquet, Agnes Tallet, Anthony Goncalves, Sébastien Lavandier, Franck Jandard, Didier Tonneau, Julien Darreon

**Affiliations:** ^1^ Aix Marseille Université, CNRS, CINaM, UMR 7325 13288 Marseille France; ^2^ Institut Paoli‐Calmettes 13009 Marseille France; ^3^ Aix Marseille Université, CNRS, UMR 7258, INSERM, UMR 1068, CRCM 13009 Marseille France

**Keywords:** inorganic scintillator detector, micro‐scintillators, real‐time radiation dose, small field irradiation, X‐ray detector

## Abstract

**Purpose:**

Small field dosimetry for radiotherapy is one of the major challenges due to the size of most dosimeters, for example, sufficient spatial resolution, accurate dose distribution and energy dependency of the detector. In this context, the purpose of this research is to develop a small size scintillating detector targeting small field dosimetry and compare its performance with other commercial detectors.

**Method:**

An inorganic scintillator detector (ISD) of about 200 µm outer diameter was developed and tested through different small field dosimetric characterizations under high‐energy photons (6 and 15 MV) delivered by an Elekta Linear Accelerator (LINAC). Percentage depth dose (PDD) and beam profile measurements were compared using dosimeters from PTW namely, microdiamond and PinPoint three‐dimensional (PP3D) detector. A background fiber method has been considered to quantitate and eliminate the minimal Cerenkov effect from the total optical signal magnitude. Measurements were performed inside a water phantom under IAEA Technical Reports Series recommendations (IAEA TRS 381 and TRS 483).

**Results:**

Small fields ranging from 3 × 3 cm^2^, down to 0.5 × 0.5 cm^2^ were sequentially measured using the ISD and commercial dosimeters, and a good agreement was obtained among all measurements. The result also shows that, scintillating detector has good repeatability and reproducibility of the output signal with maximum deviation of 0.26% and 0.5% respectively. The Full Width Half Maximum (FWHM) was measured 0.55 cm for the smallest available square size field of 0.5 × 0.5 cm^2^, where the discrepancy of 0.05 cm is due to the scattering effects inside the water and convolution effect between field and detector geometries. Percentage depth dose factor dependence variation with water depth exhibits nearly the same behavior for all tested detectors. The ISD allows to perform dose measurements at a very high accuracy from low (50 cGy/min) to high dose rates (800 cGy/min) and was found to be independent of dose rate variation. The detection system also showed an excellent linearity with dose; hence, calibration was easily achieved.

**Conclusions:**

The developed detector can be used to accurately measure the delivered dose at small fields during the treatment of small volume tumors. The author's measurement shows that despite using a nonwater‐equivalent detector, the detector can be a powerful candidate for beam characterization and quality assurance in, for example, radiosurgery, Intensity‐Modulated Radiotherapy (IMRT), and brachytherapy. Our detector can provide real‐time dose measurement and good spatial resolution with immediate readout, simplicity, flexibility, and robustness.

## 1 Introduction

Radiation dosimetry plays a very important role in radiotherapy to accurately measure the exact radiation dose delivered to the patients to ensure a high treatment quality assurance.^1^ To enhance treatment efficiency, several radiotherapy techniques have been developed for cancer treatment modalities such as intensity‐modulated radiotherapy (IMRT), volumetric modulated arc therapy (VMAT), stereotactic body radiation therapy (SBRT), and stereotactic radiosurgery (SRS) etc. These techniques require small irradiation field sizes and high dose spatial gradients to ensure the delivery of accurate high doses with tighter margins around the targeted tumors, enabling possible sparing of organs at risk.^2^, ^3^, ^4^ Unfortunately, accurate measurements are usually hampered due to the size of the conventional dosimeters^5^, ^6^, ^7^ for proper dose distribution in the treatment planning system and patient quality control.

Due to the lack of charged particle equilibrium, chamber size, dose perturbation, corrections of volume averaging effects, and nonequivalence material regarding soft tissue, the measurement with these conventional detectors is complex and hence requires many correction factors especially in the small field dosimetry.^7^, ^8^ Hence, several international organizations such as AAPM and IAEA suggested various dosimetry sensors when working under small fields. Some recent researches indicate that the suitable detectors for small field dosimetry are plastic scintillation‐based exradin W1, W2, and radiochromic films, owing to their good correction factor.^9^, ^10^, ^11^, ^12^, ^13^, ^14^ However, the spatial resolution of these detectors is not yet up to the mark due to the minimum size of the sensor head requirement and radiochromic films suffer from time consuming techniques while being used. Furthermore, the major drawback of using plastic scintillator‐based detectors is their high sensitivity to Cerenkov radiation (known as “stem” effect) observed when charge particles generated within the fiber at high energy are slowed down in the fiber core, producing a strong Cerenkov luminescence.^15^, ^16^, ^17^, ^18^

Hence, we developed an inorganic scintillator detector (ISD) based on a scintillating inorganic cluster optically coupled with a silica optical fiber, very promising on real‐time dosimetry.^18^, ^20^ Under irradiation, the cluster emits visible light that is driven through the optical fiber toward a photon counter. The detector has been tested for different dosimetric parameters under high‐energy beams of 5–15 MV and experimental conditions close to the real patient treatment scenario. In this context, the aim of this research work is to demonstrate the performances of this ISD detector by quantitative comparison with microdiamond dosimeter (a suitable detector for small fields^8^, ^19^). PinPoint three‐dimensional (PP3D), another commercial dosimeter used for regular beam in the patient treatment planning, was also considered to show the behavior of ISD with respect to it. Lateral profiles of small and very small size fields (ranging from 3 × 3 cm^2^ down to 0.5 × 0.5 cm^2^) as well as percentage depth dose (PDD) were systematically performed with all the detectors.

## 2 Materials and Methods

### 2.1 Source

Experiments were performed under a LINAC (Elekta Synergy, VERSA) source with the photon beam energy of 6 and 15 MV at the Institute Paoli‐Calmettes (IPC), Marseille, France. The MLC window of the LINAC can be opened down to a length of few millimeters . Elekta LINAC Synergy system can deliver a typical dose rate at 400 MU/min and VERSA can work in two modes of operation (with flattening filter and without flattening filter) in a very high dose rate of 2400 MU/min. The radiotherapy equipment is periodically calibrated so that 1_MU corresponds to 1_cGy under reference condition (IAEA TRS 381). The source can irradiate fields at 40 × 40 cm^2^, down to 0.5 × 0.5 cm^2^ at the isocenter (100 cm from the source). The Elekta system can be rotated up to 360^0^ during irradiation process.

### 2.2 Devices

**Figure 1:**
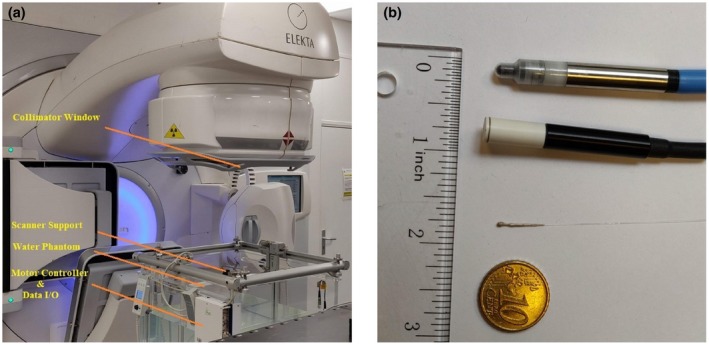
(a) Elekta Linear Accelerator source equipped with a PTW^TM^ motorized three‐dimensional (3D) water phantom. (b) Inorganic scintillator detector head dimension (below) compared with microdiamond (middle) and PinPoint 3D (top) dosimeters. [Color figure can be viewed at http://wileyonlinelibrary.com]

The novel X‐ray probe consists of a 10 ‐m‐ long silica (SiO_2_) optical fiber with scintillator clusters grafted at one terminal. Under irradiation, the scintillating clusters fixed at the fiber extremity produce visible light proportional to the irradiation coming from the LINAC source. The visible photons are transmitted through the fiber core toward a photon counter (Aurea^TM^). The core and cladding diameter of the fiber is respectively, 100 and 125 μm (Thorlabs^TM^), and the fiber bandwidth is 400–2100 nm. The scintillating material, ZnS:Ag, is a powder of 2–3 µm grain size typically, mixed with PMMA resist diluted in Ethyl lactate (C_5_H_10_O_3_) solvent. PMMA is a biocompatible resist commonly used in microelectronics industry. After removing the plastic protective coating from glass optical fiber, the extremity is dipped into this PMMA mixture and immediately removed for a drying step at 65°C. Consequently a nearly spherical PMMA droplet containing ZnS:Ag clusters is formed. Finally, the device is dipped in a liquid silver paste and dried at room temperature. This latter metallization step guided the device to be free from any ambient light noise. The head of this detector in comparison to other conventional dosimeters is shown in Fig. 1(b). The sensitive volume is assumed to be a cylinder of diameter 100 µm (fiber core diameter) and 1.5 µm in length. Indeed, light emitted by scintillating grains at distances higher than 1.5 µm from the fiber core is re‐absorbed before reaching the core. Thus, we estimate that the sensitive volume of ISD is about 1.2 × 10^−5^ mm^3^. This sensitive volume is much lower than that of PP3D (0.016 cm^3^ with an outer diameter of 7 mm) and microdiamond (0.004 mm^3^ with an outer diameter of 4.8 mm).

### 2.3 Experimental set‐up

Figure 1 represents the detailed experimental setup used with the LINAC Elekta source and the water phantom. This system is based on a largesize water tank (IBA^TM^) equipped with X, Y, and Z stages that allows dose distribution measurements in arbitrary planes, according to international standards such as AAPM TG 142. Results presented in this paper were obtained by keeping the beam perpendicular to the water surface and moving the sensor in planes parallel to this surface. The scintillating active part of the fiber‐based detector is fixed to the scanning unit inside the water tank, whereas the other extremity of the fiber is plugged to a photon counter. The whole setup is remotely controlled from an external room avoiding any exposure of the electronics to high‐energy irradiation.

### 2.4 Measurement protocol

To demonstrate the sensor performance, all the measurements were carried out simultaneously with a microdiamond dosimeter commonly used for small field dosimetry and a PP3D detector. Unless otherwise stated, each measurement in this research has been measured inside water phantoms in reference conditions, that is, SSD (source to water surface distance) of 90 and 10 cm depth in water during beam profiling and 100 cm SSD during PDD measurements. The sensitive photon counter measures the optical signal of the scintillation light in photons per second. Time integration of optical signal gives the total number of photons linked to the irradiation dose. A Matlab^TM^ simulator was developed to calculate the total amount of photons during each irradiation. In order to compare the performance of the scintillator detector with other dosimeters, beam profiles and PDD curves have been normalized.

### 2.5 Cerenkov light subtraction

The spectral distribution of Cerenkov light is most intense in the blue and ultraviolet regions of the electromagnetic spectrum. This effect has a huge contribution on signal amplitude when using plastic scintillators and optical fibers.^15^, ^21^, ^22^, ^26^ Thus, the Cerenkov effect in this case must be considered and removed from the total acquisition signal. In our case, the inorganic scintillator is grafted to a narrow silica fiber core, so that the Cerenkov effect is expected to be weak. Moreover, in order to minimize this effect, the size of the inorganic scintillating head has been reduced as much as possible.^21^, ^22^ However, even if the contribution of the Cerenkov Effect is weak, it was systematically quantitated and removed from the measurements presented in this article. A background fiber method^15^, ^23^, ^24^ has been used to accurately measure the real scintillating light. Indeed, the detector and a bare fiber were simultaneously exposed to radiation at the same x‐position [Fig. 1(a)]. It relies on the assumption that the Cerenkov signal generated in the background fiber is of equal magnitude as the signal fiber. Finally, the actual signal of the detector is obtained by subtracting the signal of the bared fiber from the signal provided by the detector directly read from the photon counter. This Cerenkov correction was considered for all the recorded data given in this study.

## 3 Results

### 3.1 Relative dose measurement

**Figure 2:**
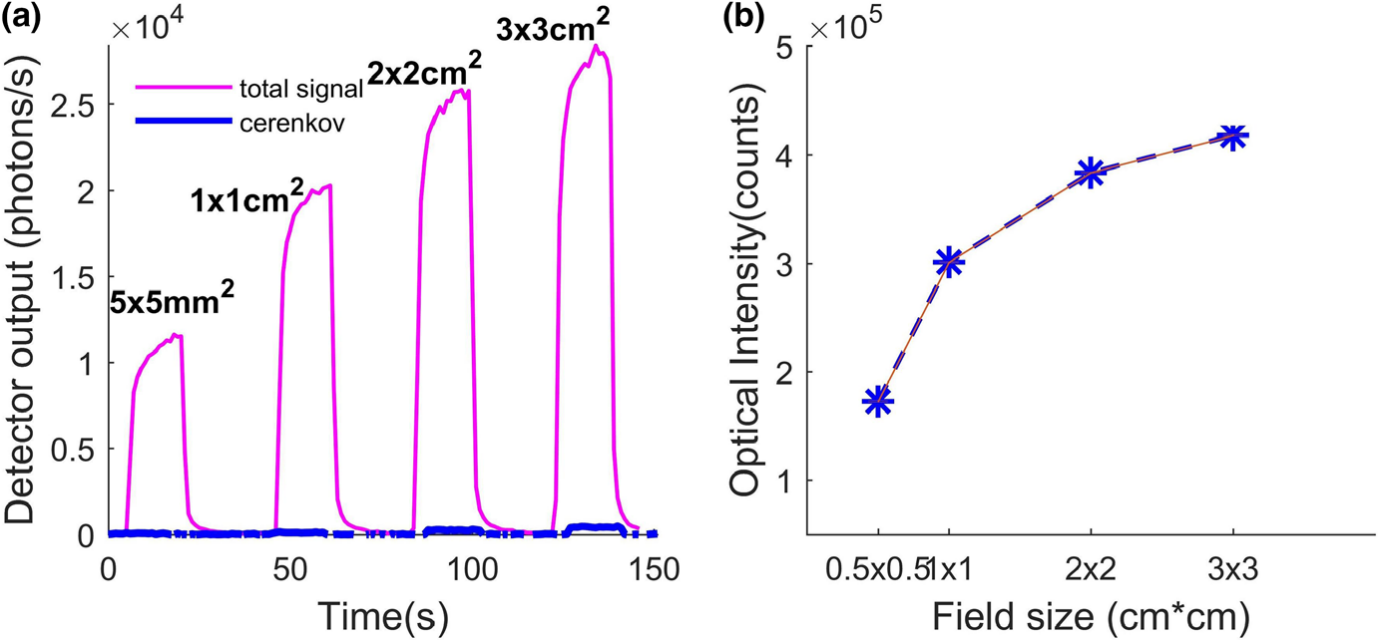
(a) Inorganic scintillator detector output signal with time for different fields of 100 MU doses delivered at 400 MU/min. (b) Integrated output signal as a function of field size. [Color figure can be viewed at http://wileyonlinelibrary.com]

Figure 2(a) shows the optical signal magnitude variation with time and respective field sizes (ranging from 3 × 3 cm^2^ down to 0.5 × 0.5 cm^2^) at 6 MV. These measurements were performed with the sensor placed at the field center in reference conditions. For each field, the acquisition signal was recorded during the delivery of 100 MU dose. Each curve exhibits the same behavior with a rise time of 5 s followed by a plateau. Finally, a fall time in the microsecond range is observed when the beam is switched off automatically. The photon counter used in this study has a rise and fall time in the ns range (constructor data), so the long raise time demonstrates the LINAC source characteristic. Figure 2(a) also shows the Cerenkov effect magnitude (blue lines) under the same irradiation conditions. As mentioned in Section 2.E, this effect has a very low contribution to the signal recorded by our detector and can be easily eliminated from the total signal. Finally, the total number of photons (counts) corresponding to the actual scintillation during irradiation is calculated by integrating the optical signal (free from Cerenkov) with respect to time. This optical signal intensity reported in Fig. 2(b) increases with the field size, as the diffusion inside the multileaf collimator is increasing. Due to the very low noise level (300 photons/s) and low signal fluctuations (<0.2%) during irradiation signal measured (about 10^5^ photons/s), error bar of each ISD measurement is within the dot size on the curve shown in Fig. 2(b).

### 3.2 Dose repeatability and reproducibility

**Figure 3:**
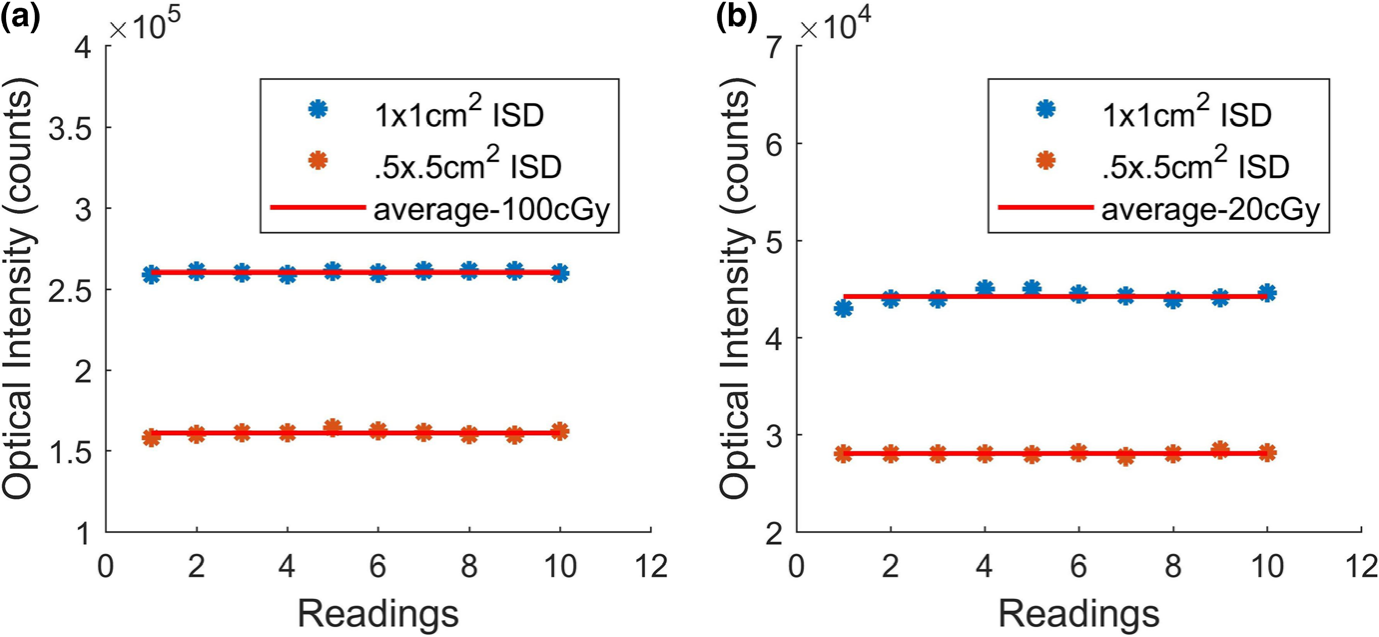
(a) Repeatability of measurement with inorganic scintillator detector (ISD) for ten successive irradiations at 100 MU. (b) Repeatability of measurement with ISD for ten successive irradiations at 20 MU. [Color figure can be viewed at http://wileyonlinelibrary.com]

Measurement repeatability was checked under standard conditions of 15 MV for 100 MU [Fig. 3(a)] and 20 MU [Fig. 3(b)] doses, delivered at a dose rate of 400 MU/min. For this test, ten consecutive irradiations were performed on the sensor placed at the center of 1 × 1 cm^2^ and 0.5 × 0.5 cm^2^ fields.

These results show that the ISD demonstrates a very good repeatability with a maximum standard deviation of 0.26% and 1.2% from the average doses of 100 and 20 cGy, respectively. The detector was also tested for seven consecutive days and <0.5% day‐to‐day variation of the collected signal was observed. This result demonstrates a very good reproducibility and highlights that ISD does not require regular calibration.

### 3.3 Dose and dose rate linearity

**Figure 4:**
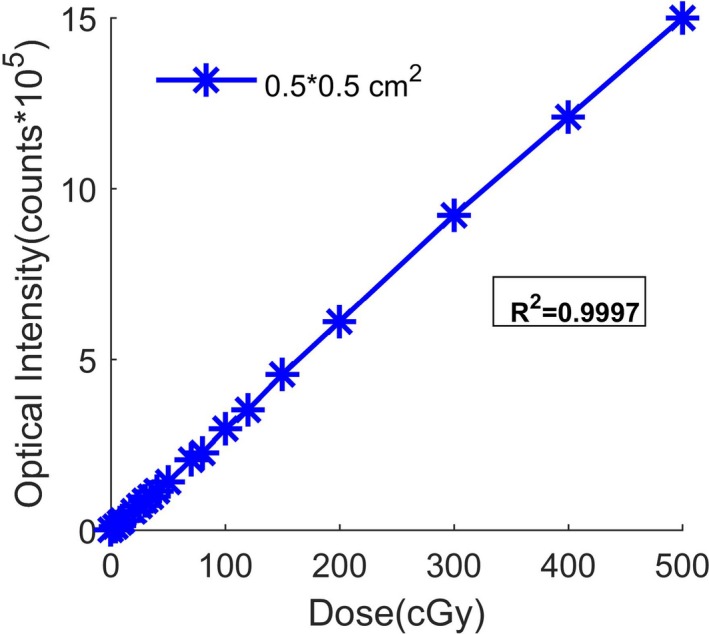
Inorganic scintillator detector signal linearity with dose. [Color figure can be viewed at http://wileyonlinelibrary.com]

**Figure 5:**
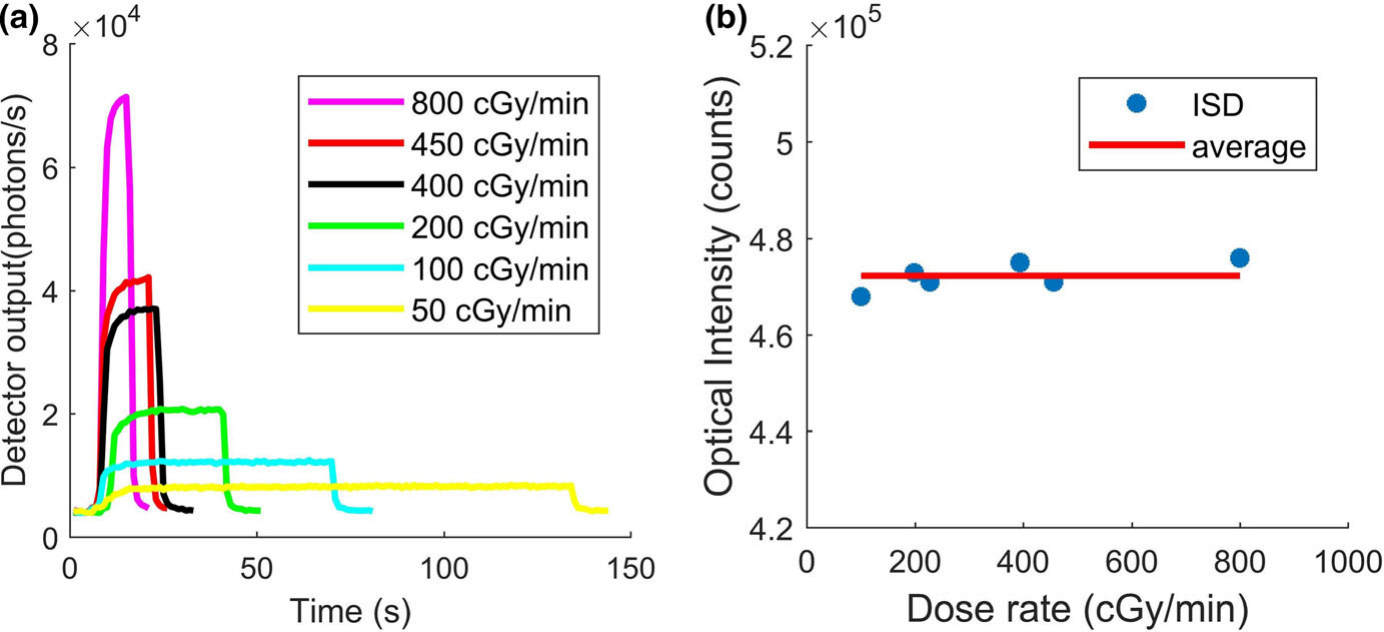
(a) Variation in signal as a function of time for 100 cGy delivered with dose rates ranging from 50 to 800 cGy/min. (b) Integrated number of visible photons generated by the inorganic scintillator detector corresponding to Fig. 5(a). [Color figure can be viewed at http://wileyonlinelibrary.com]

Detector’s linearity was tested from very low dose (5 cGy) to high dose (500 cGy) at the center of smallest field and observed accurate linear behavior with a linear regression factor of 0.9997, as can be seen in Fig. 4. Figure 5(a) shows the variation in the optical signal measured with ISD as a function of time for different dose rates ranging from 50 to 800 cGy/min. The total dose was kept constant at 100 cGy for each experimental measurement point. As evident, the higher the dose rate, the shorter is the irradiation period. Figure 5(b) represents the variation in the total number of photons measured during each irradiation as a function of dose rate. As expected, the detector provides an optical signal that is almost independent of dose rate. Indeed, the maximum standard deviation of the signal from the average value is as low as 0.15%, which is a very significant outcome when compared to that obtained with other detectors [see, e.g., 14, 15, 25]. This result shows that ISD can be successfully used at a low as well as a high dose rate, as it is important for a broad range of radiotherapies.

### 3.4 Beam profiling and comparison

**Figure 6:**
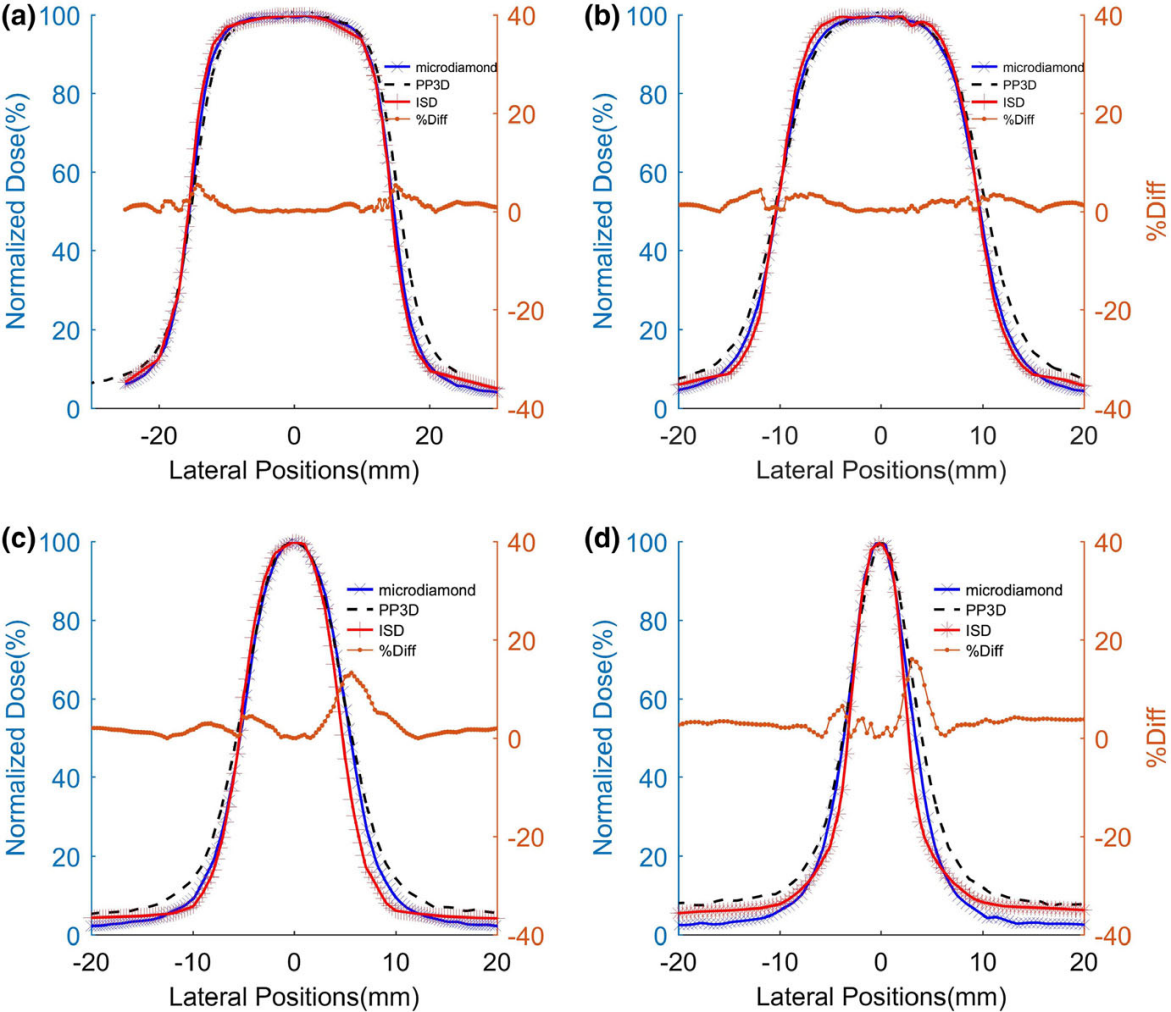
Lateral beam profiles obtained using inorganic scintillator detector (ISD) (red), Microdiamond (blue line) and PinPoint three dimensional (black dashed line) for (a) 3 × 3 cm^2^, (b) 2 × 2 cm^2^, (c) 1 × 1 cm^2^ ,and (d) 0.5 × 0.5 cm^2^ field sizes. The brown dashed line shows the percentage difference in the measurements between ISD and microdiamond. [Color figure can be viewed at http://wileyonlinelibrary.com]

Beam profiles were measured for fields ranging from 3 × 3 cm^2^‚ down to 0.5 × 0.5 cm^2^ with ISD, PP3D, and microdiamond dosimeters in reference conditions inside water phantoms. We have reported in [Figs. 6(a) to 6(d)], the normalized local dose as a function of detector position in crosslink within the field. The step size between two successive measurements is maintained 200 μm. The in‐field percentage difference shows only (0–1.2)% variation with respect to the reference detectors, which is in a very good agreement. The profiles shown in Fig. 6 exhibit a nearly sharp falloff at the field edges, as expected for all detectors. Indeed, because of scattered radiation and convolution effect between field and detector geometries,^27^, ^28^, ^29^ a perfect falloff cannot be achieved. Both effects are usually gathered in the penumbra region. Convolution effect contribution is roughly proportional to the detector sensitive head size. In our case, thanks to the small size of ISD, the penumbra profile (between 20–80%) is sharper in comparison to the other dosimeters considered in this research.

Using ISD, the measured full width half maximum (FWHM) is 3, 2, 1, and 0.55 cm, which is in very good agreement with the selected beam size. Little discrepancies between ISD and the microdiamond detector at field edges are observed at a level around 4.45%–13.3%.

### 3.5 PDD measurements and comparison

**Figure 7:**
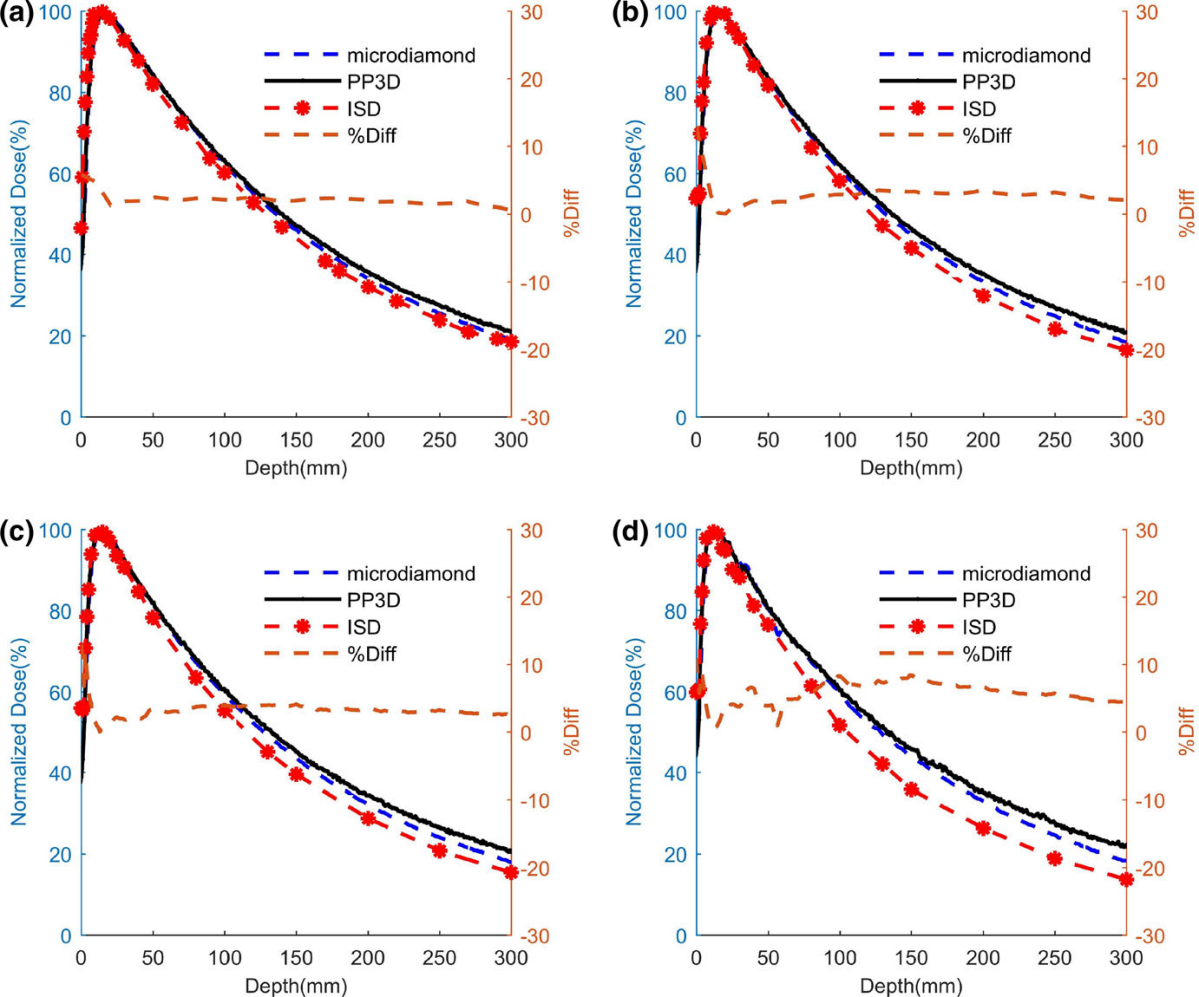
Percentage depth dose measurements obtained using inorganic scintillator detector (ISD) (red) compared with the microdiamond (dashed dark blue line) and PinPoint three‐dimensional (black) for (a) 3 × 3 cm^2^ (b) 2 × 2 cm^2^, (c) 1 × 1 cm^2^ and (d) 0.5 × 0.5 cm^2^ fields. The brown dashed line shows the difference between measurements given by ISD and microdiamond. [Color figure can be viewed at http://wileyonlinelibrary.com]

The central axis dose distribution is one of the most important clinical parameters and it is typically characterized by PDD factor measurements. The PDD curves obtained with ISD are presented in Fig. 7 for different small fields varying 3 × 3 cm^2^ down to 0.5 × 0.5 cm^2^ and comparison were shown with microdiamond detector. Due to the increasing number of scattered photons and secondary electrons created by the incident high energy beam penetration, the PDD steeply increases till maximum depth dose in the buildup region. After the maximum value, the PDD smoothly decreases with the depth due to the absorption of generated photons and/or charged particles by water. With ISD, the maximum PDD value is found at 15 mm from the water surface, a standard and expected value for the 6‐MV incident photon beam.^15^, ^27^, ^28^, ^30^ This maximum dose depth is independent of the field size considered here. A very good agreement is found between the measurements given by the three detectors for 3 × 3 cm^2^ and 2 × 2 cm^2^ fields. However, far from the maximum dose depth, differences between detectors become visible and are more pronounced at small fields.

The PDD difference between ISD and microdiamond detector varies from 0.1 to 13.3%, whereas the average percentage difference stays in a reasonable range, from 2.1 to 5.6% for all the measurements. Note that, the PDD difference around the maximum of the buildup region stays below 1% for all the small fields.

## 4 Discussion

The developed ISD sensor detects a maximum dose delivery at a depth of approximately 15 mm inside water, a value in perfect agreement known to radiotherapy dosimetry. This value for maximum dose is independent of the field size from 3 × 3 cm^2^ down to 0.5 × 0.5 cm^2^. The PDD exhibits almost the same behavior for all detectors unless a little discrepancy is observed for ISD, especially in water depths larger than 15 mm. This effect can be strongly reduced by necessary corrections of the reference detector.^31^, ^32^, ^33^ Moreover, some discrepancies that appear in small fields and nearby water surfaces can be due to the proper centering of the reference dosimeter. However, discrepancies observed at a higher depth can be attributed to the different sensitivities of each detector to incident and secondary photons as well as other charged particles.

The ISD detector has a very small scintillating active part (1.2 × 10^−5^ mm^3^) and thus allows to measure more accurately dose distribution at the edge of the beam profile. Indeed, the minimum step size of 200 µm available in the scanning unit (IBA^TM^) was selected to characterize all the beam profiles.

For the 0.5 × 0.5 cm^2^ field, we measured an experimental FWHM of 0.55 cm. The small discrepancy between expected and measured field sizes can be explained by three different effects. First, the measured field is wider due to scattered photons on the edge of the thick lead collimator of the LINAC. Secondly, the field also expanded due to charged particle generation inside water in the detector surface vicinity. Finally, the convolution effect between the actual field geometry (square of 5 mm side) and the detector geometry must be considered. This latter effect leads to an apparent side increase in 100 µm, that corresponds to the ISD sensor diameter.

The minimum step available on PTW translation stage is 200 µm. In these conditions, the signal difference between two consecutive measurements in the penumbra region is about 4000 photons/s, which is much higher than the photon counter sensitivity (20 photons/s). Thus, the step size could be decreased down to 100 µm and even less using a lower diameter core fiber keeping a significant signal to noise ratio. Note that, we already presented a few µm lateral resolution ISD, designed for low‐energy (2–30 keV) X‐ray beam profiling.^34^ In this study, we found a detection flux threshold as low as 10^3^ X‐ray photons/s/μm^2^. Thus, the design of an ISD detector for a high‐energy beam offering a spatial resolution much better than 100 µm is realistic.

In contrast to the plastic scintillating detector (PSD), Cerenkov contribution in the developed detector was found to be negligible regarding the magnitude of the optical signal measured in the experimental conditions. Moreover, the ambient optical noise coming from the experimental room was avoided by coating the sensor head with a very thin metallic layer embedding the scintillators. Thus, the scintillating detector in this regard exhibits a higher signal to noise ratio.

Device stability and reproducibility tested at various dose rates ranging from 50 to 800 cGy/min will allow to use it at low dose application for small size tumor and high dose delivery in brachytherapy and radiosurgery. ISD is based on optical transitions that are less sensitive to ambient temperature and pressure variations than electrical charge‐based detectors. Therefore, it is anticipated that the ISD detector is free from any perturbations due to temperature and pressure variations. This result is still under investigation by relevant experimentation.

Finally, the ISD provides 4.75*10^5^ visible photons collected with the photon counter for a total dose of 100 cGy, independently of the dose rate (Fig. 5). The calibration is thus about 4700 visible photons generated per cGy of dose delivered. As the detector’s signal magnitude is proportional to the dose, this system can easily be used on patients for radiotherapy dose measurement for small field irradiation with better accuracy.

## 5 Conclusions

Due to the tiny scintillating sensitive volume used at the optical fiber extremity (about 1.2 × 10^−5^ mm^3^), the developed detector in this article exhibits a very high lateral resolution in dosimetry measurements. Indeed, it allows to accurately define the lateral profiles of very small fields down to 0.5 × 0.5 cm^2^ with a precision of around 200 µm. Owing to the detector’s minimum size (nearly an ideal point detector), ISD is almost free from detector edge effects and no aberrant measurements were observed, while it was the case for the concurrent microdiamond and PP3D detectors. The high spatial resolution of the ISD was examined during the sharp falloff in the penumbra region of the smallest beam profiling in comparison with the commercial detector.

The real‐time inorganic detector in this study is fast, robust, and nonsensitive to external noise and stem effect. As expected, the integrated total relative dose measurement in this study was found to be independent of the dose rate variability. Moreover, a perfect linearity with dose was observed for the smallest field considered in this study and lead to detector calibration of 4.7 10^5^ photons collected/Gy.

Further analysis on detector characterization and resolution improvement is ongoing, whereas the results presented in this research demonstrate the prospects of ZnS:Ag–based scintillator detector. The performance of ISD shown in this study demonstrates that the detector can be a suitable candidate for small field dosimetry, a proper quality control tool for possible early stage tumor treatment. However, further investigation of the detector must follow the comparison with water equivalent scintillating dosimeter, for example, PSD, radiochromic films etc., during absolute dose measurements.

## Conflict of Interest

The authors have no conflict of interest to disclose.
